# War and peace: social interactions in infections

**DOI:** 10.1098/rstb.2013.0365

**Published:** 2014-05-19

**Authors:** Helen C. Leggett, Sam P. Brown, Sarah E. Reece

**Affiliations:** 1 Department of Zoology, Oxford University, South Parks Road, Oxford OX1 3PS, UK; 2 Biosciences, University of Exeter, Cornwall Campus, Penryn TR10 9EZ, UK; 3 Institute of Evolutionary Biology, University of Edinburgh, Edinburgh EH9 3JT, UK; 4 Centre for Immunity, Infection and Evolution, School of Biological Sciences, Ashworth Laboratories, University of Edinburgh, Edinburgh EH9 3JT, UK

**Keywords:** plasticity, transmission, virulence, relatedness, ecology, inclusive fitness

## Abstract

One of the most striking facts about parasites and microbial pathogens that has emerged in the fields of social evolution and disease ecology in the past few decades is that these simple organisms have complex social lives, indulging in a variety of cooperative, communicative and coordinated behaviours. These organisms have provided elegant experimental tests of the importance of relatedness, kin discrimination, cooperation and competition, in driving the evolution of social strategies. Here, we briefly review the social behaviours of parasites and microbial pathogens, including their contributions to virulence, and outline how inclusive fitness theory has helped to explain their evolution. We then take a mechanistically inspired ‘bottom-up’ approach, discussing how key aspects of the ways in which parasites and pathogens exploit hosts, namely public goods, mobile elements, phenotypic plasticity, spatial structure and multi-species interactions, contribute to the emergent properties of virulence and transmission. We argue that unravelling the complexities of within-host ecology is interesting in its own right, and also needs to be better incorporated into theoretical evolution studies if social behaviours are to be understood and used to control the spread and severity of infectious diseases.

## 1. Introduction

Social acts, ranging from minor help to major self-sacrifice, are seen in all walks of life, from humans to microorganisms. It used to be generally assumed that the parasites and microbial pathogens that cause infectious diseases lived relatively independent unicellular lives, without the cooperative behaviours that have provoked interest in mammals, birds and insects [1]. However, a rapidly expanding body of research demonstrates that much of what parasites and microbial pathogens do, they do in groups. Furthermore, parasites and microbial pathogens display some amazing natural history, including behaviours described as mafia strategies, body-snatching, chemical warfare, mass suicide, suicide bombing and weapons of mass destruction (reviewed by [2–15]).

The expanding interest in understanding social evolution in parasites and microbial pathogens has probably occurred for two reasons. First, they are often well-described and tractable experimental systems for studying the ecology and evolution of social traits in real time, under both highly controlled conditions and in a ‘real-world context’, which for pathogens and parasites involves being exposed to the complex, changeable and hostile environments inside a host or vector. Second, sociality is a driver of the damage pathogens and parasites do to their hosts (virulence) [16,17], it shapes survival of medical interventions (such as antibiotics) [18,19], and underpins between-host transmission (e.g. [20,21]). Thus, examining the behaviours of parasites and microbial pathogens from the perspective of ‘a life in society’ is one of the most important issues in applied evolutionary biology. However, the mechanisms through which parasites and microbial pathogens interact with each other, the host/vector and the abiotic environment has been largely overlooked within the evolutionary/ecological search for general principles (and their empirical support) of the often-connected theories for inclusive fitness and virulence evolution.

It is our view that a ‘bottom-up’ approach to studying microbial pathogens and parasites is the next milestone for understanding their social behaviours and for controlling the infectious diseases they cause. In this article, our aims are to showcase recent empirical and theoretical work demonstrating that a biologically informed bottom-up view: (i) illustrates the extremely rich phenotypic landscape of parasites and microbial pathogens at the within-host scale; (ii) enables integration across levels of biological organization, from the molecular mechanisms underpinning social behaviours to population ecology, to capture the biological complexity required to explain social systems; (iii) can provide novel insight into the evolution and ecology of social behaviours in general; and (iv) offers novel approaches to disease control with the potential to be more ‘evolution-proof’ than current therapies.

We also wish to facilitate cross-discipline communication between empirical and theoretical evolutionary ecologists and biologists in more applied disciplines such as microbiology, parasitology and biomedicine. To achieve this, we begin by providing an overview of the basic evolutionary and ecological frameworks for how social behaviours are studied (§2) and why virulence evolves (§3). The aim of these sections is to furnish readers unfamiliar with the social evolution and virulence evolution literatures with the concepts underpinning the recent developments that form the focus of the following sections. Therefore, aficionados in ecology may wish to skip to §4. The figures and tables illustrate the concepts we discuss in the text. Most examples concern malaria (*Plasmodium*) parasites and microbial pathogens (bacteria and bacteriophage) because these groups span the taxonomic diversity of infectious disease causing organisms, and together they offer the opportunity to integrate understanding at multiple levels of biological organization, from genes and molecular pathways, to phenotypes, to epidemiology [22,23]. For brevity when discussing general concepts, we collectively refer to parasites and microbial pathogens as ‘parasites’ owing to their shared lifestyle of exploiting hosts.

## 2. Social behaviours

All organisms interact with others throughout their lives, including with family members, unrelated conspecifics and hetero-specifics. Social interactions range from extreme conflict (e.g*.* lethal combat) to extreme cooperation (e.g*.* altruistic suicide or sterility) but most interactions lie somewhere between these extremes. Social behaviours can be categorized according to their impact on the lifetime reproductive success of the ‘actor’ expressing a particular social phenotype and any ‘recipients’ impacted by the actor's phenotype (table 1) [24,48–50]. Taking a simple +/− dichotomy for both actor and recipient gives a simple four-part categorization: (i) *mutual benefit* (+/+), where the actor and recipient both gain from the actor's behaviour; (ii) *selfishness* (+/−), where the actor gains at the expense of the recipient; (iii) *altruism* (−/+), where the behaviour is detrimental to the actor but beneficial for the recipient and (iv) *spite* (−/−), where the behaviour is harmful for both actor and recipient. The pioneering work of Bill Hamilton [24,48] provided a foundation to explore how natural selection drives the spread of these four types of social behaviours through a population. The topics outlined below illustrate the key concepts involved in social interactions.

**Table 1.** A classification of social behaviours, after [24–26]. These examples illustrate that the richness of social behaviours observed in multicellular organisms are mirrored in parasites. Moreover, parasite social behaviours often have consequences for the severity and transmission of disease. Note that it is extremely difficult to quantify costs and benefits of many social behaviours, for actors and recipients, so many of these examples are yet to be fully understood.

		effect on recipient	
		+	−
effect on actor	+	mutual benefit	selfishness
		*Multicellular taxa.* This ranges from simple scenarios such as group cooperation providing safety in numbers, to rewarding helpers or punishing non-helpers. Group activities in the naked mole rat, *Heterocephalus glaber*, reduce the risks of predation, hypothermia and starvation [27–29]. Unrelated subordinate *Polistes* wasps cooperate to raise their colony's offspring because there is reasonable chance of inheriting the dominant's breeding position in the near future [30]. In this case, helpers ‘pay to stay’ because the acquisition of breeding opportunities is a large pay-off. *Parasites*. The release of secreted public goods molecules into a sufficiently small and/or structured population can generate mutual benefits, so long as the fraction of reward returned to the actor cell exceeds the direct costs of production, and that some reward is felt by cells other than the actor. Positive interactions between species have been documented. An existing infection of *Babesia microti* in field voles (*Microtus agrestis*) appears to increase the probability of *Anaplasma phagocytophilum* infection and vice-versa [31]. Also, if mixed infections are more challenging than single infections for host immune responses, mutual benefit could occur, which may result in greater virulence.	*Multicellular taxa.* The classic example is male lions killing their predecessor's cubs when they take over a pride [32]. This brings lionesses into season and so hastens the new male's mating opportunities. Selfish acts can also be disguised as cooperation; white-winged choughs cheat by attempting to fool dominants that they are helping at the nest [33]. *Parasites*. Cheating is rife in bacterial infections; the cost of producing extracellular iron-scavenging siderophores in *Pseudomonas aeruginosa* selects for non-producing cheats who can outcompete cooperators [34,35]. Because cooperation is required to efficiently acquire the iron, mixed infections can be less virulent [36]. By contrast, competition in co-infections selects for faster replication in *Plasmodium* and phage, which causes greater virulence to the host [37,38].
	−	altruism	spite
		*Multicellular taxa*. The classic example is the eusocial insects (including bees, ants, termites) in which a single queen (or few) monopolize the colony's reproduction [39]. All others in the colony are morphologically or behaviourally specialized to altruistically forage, guard or raise offspring. *Parasites*. Morphological castes have been described in *Philophthalmus* sp. trematodes, which produce reproductive and non-reproductive morphs. The sterile morph increases the reproductive output of relatives but only when competing strains share the host [40]. Other examples include suicide in *Salmonella typhimurium* to facilitate the gut invasion of others [41], and in *Escherichia coli* infected with phage to prevent parasite transmission to others [42]. Forms of suicide have been described in *Plasmodium* and trypanosomes, which may regulate density, preventing premature death of the host/vector [15,43]. Suicidal release of a public good is not a necessary condition for altruism—the release of a costly public good into a dense and well-mixed population (minimizing the direct return to the actor cell) is likely to be an altruistic act.	*Multicellular taxa*. Soldiers of the polyembryonic parasitoid wasp *Copidosoma floridanum,* are sterile because they never reach sexual maturity. They preferentially kill other, unrelated embryos developing in their host, which frees up resources for their siblings [44]. Workers of the red fire ant *Solenopsis invicta*, kill related queens who do not have a greenbeard gene, ensuring that the greenbeard gene persists [45]. *Parasites*. Bacteria secrete anti-competitor toxins that are costly to produce (e.g. toxin release requires cell lysis in many cases). Toxin production and immunity genes are usually linked so that relatives are immune. Virulence is lower in mixed infections in caterpillar hosts compared with single infections when competing strains are susceptible to each other's bacteriocins [46]. However, the impact of spite on virulence becomes more complicated when spiteful behaviours are affected by simultaneous investments in other social traits (e.g. public goods) [47].

### (a) Inclusive fitness: all for one and one for all

Hamilton's key insight was that genes controlling the social traits of an actor can influence the replication of gene-copies in recipients. In the case of altruistic traits, Hamilton's logic reveals a simple genetic nepotism—helping neighbours is another way of helping your own genes to reproduce, so long as they carry the helper-genes of interest. Hamilton proposed a critical metric to weight the likelihood that recipients carry the gene of interest, termed relatedness [51]. Common descent or kinship is the most common reason for interacting individuals to share genes with above-average frequency in a population. Consequently, relatedness can be understood as the chance of gene sharing among kin, above and beyond average probability [52]. Inclusive fitness partitions natural selection into direct and indirect effects; direct effects describe the impact of an individual's own genes on reproductive success, and indirect effects describe the impact of the focal individual's genes on the fitness of its social partners, weighted by genetic relatedness [24,26,48]. Cooperation may be mutually beneficial if it directly benefits the actor as well as the recipients, for example, by increasing the success of an individual's own group (table 1). More extreme acts of altruistic cooperation may be selected if the behaviour helps recipients who are very likely to share the altruistic gene (i.e. if relatedness is high such as within families) [24,26,48]; thus indirectly propagating genes for altruism. An important point to note is that many parasite species reproduce asexually (i.e. clonally) during at least one stage of their life cycle [53], and thus each group of clonally related parasites (genotype) within an infection is expected to behave as a multicellular organism [54] because the genotype is the target of selection.

### (b) Cheating: playing the system

When relatedness is low, cooperative behaviours are vulnerable to exploitation by cheats that do not contribute to collective action but still benefifit from the cooperative behaviours of others [6]. Cheats can proliferate under these conditions because the benefits of cooperation are shared indiscriminately, and consequently genes for cheating will have greater fitness than the genes for cooperation [34,55]. The spread of cheats through a population can in turn lead to a decline in population fitness (an idea encapsulated by Hardin's ‘Tragedy of the commons’ [56] and by ‘the Prisoners’ dilemma’ [57]). Empirical studies have demonstrated that cheating can indeed occur in numerous cooperative systems of microbial pathogens [7,34,58–60]. Recent years have witnessed a surge in the application of evolutionary theory to explain the ways in which cooperation is maintained (reviewed by [1,3,6,61–63]), which includes mechanisms for kin discrimination and communication.

### (c) Kin recognition: deciding who to help

**Figure 1.**
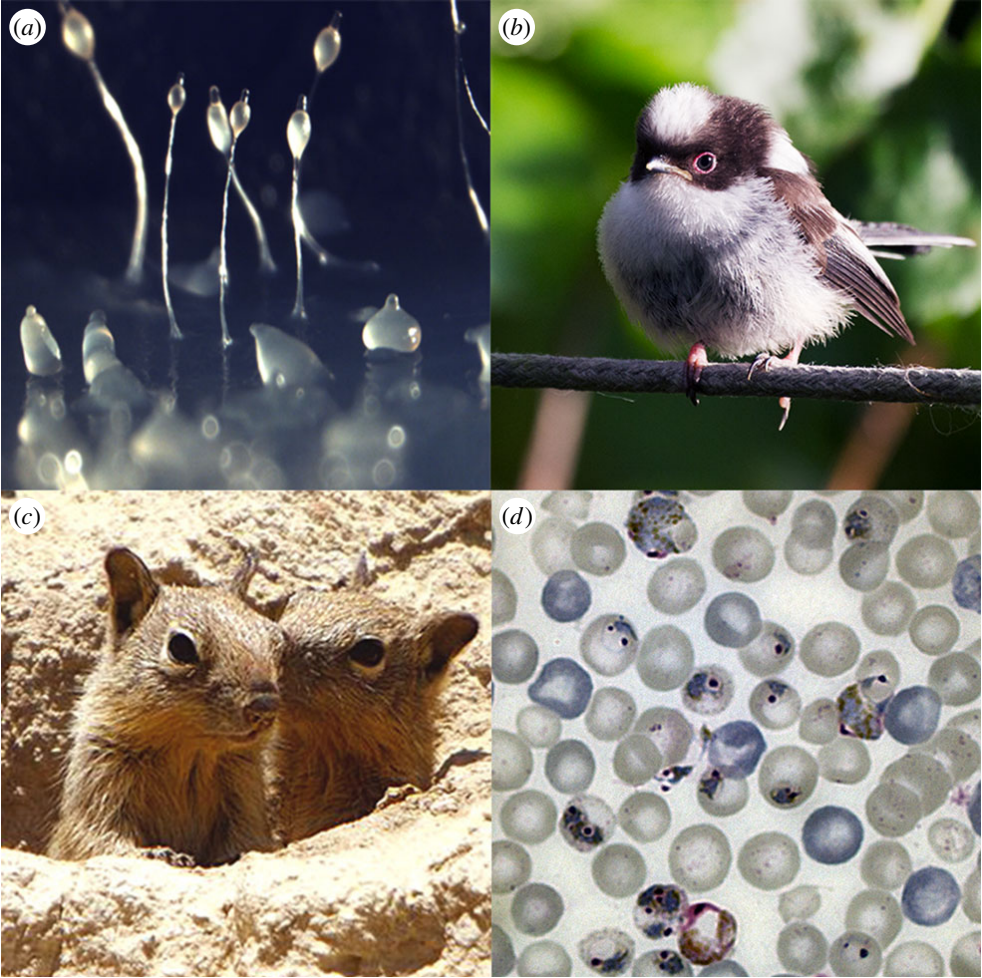
Examples of kin discrimination by: (*a*) direct recognition, e.g. cells of the slime mould *Dictyostelium* determine whether they are interacting with kin or non-relatives during slug and spore formation based on the sequence similarity of their surface adhesion proteins [66,67] (photo credit Owen Gilbert); (*b*) indirect cues based on familiarity with individuals, e.g. long-tailed tits learn the vocalization patterns of kin during the natal rearing period [68] (photo credit Sarah Reece) or (*c*) ‘armpits’ which are a mixture of direct and indirect cues, e.g. ground squirrels use olfactory cues which have a genetic component and are also learnt by self-referencing during development [69] (photo credit Alan Vernon). The malaria parasite, *Plasmodium chabaudi* (*d*), adjusts investment into male and female transmission stages according to how many other conspecific clones share the host, suggesting kin discrimination occurs [21] (photo credit Sarah Reece and Sinclair Stammers). The mechanism is unknown but indirect cues seems unlikely; an obvious candidate would be that parasites can infer the presence of other clones via the host immune response, but sex ratio adjustment is observed in infections before the required strain-specific responses develop.

Relatedness is key to understanding the direction and magnitude of selection on social traits, but what shapes relatedness? A commonly cited scenario is that social acts are expressed blindly to neighbours, who tend to be relatives simply because of incomplete mixing of individuals in populations—the population is ‘viscous’ [24,48]. However, altruists in this system may fall victim to ‘cheats’ that lack the gene for altruism. A way to avoid wasting help on cheaters is to display an altruistic or social gene and to recognize the same gene in others, and only direct help to individuals expressing that gene—a notion popularized as a ‘green beard’ [64,65]. Another way to direct cooperative behaviours towards appropriate recipients is through the ability to recognize kin (figure 1). Kin discrimination can occur via: (i) direct recognition, (ii) indirect cues that convey whether a recipient is likely to be a relative; or (iii) a mixture of direct and indirect information. Kin discrimination systems can require additional selective forces to maintain polymorphisms that can be used as accurate identifiers [70]. Host–parasite systems, in which genotype-by-genotype interactions and frequency-dependent selection maintain genetic variation, are candidate motors maintaining the genetic diversity required for kin discrimination (figure 1).

### (d) Communication: coordinating collective action

Parasites have evolved sophisticated communication systems to coordinate behaviours across clone-mates and enable collective actions to be efficiently deployed. For example, 6–10% of all genes in the opportunistic microbial pathogen *Pseudomonas aeruginosa* are controlled by cell–cell signalling systems [71]. Coordination is especially important for behaviours that must be expressed by some, but not all individuals. For example, in cases where the suicide of some individuals benefits survivors, the suicide trait must not be expressed by all individuals otherwise there would be no survivors [15,41]. Equally, undertaking costly cooperative actions may only pay when the numbers of actors exceeds a certain threshold, and so density-sensing mechanisms are often used to ensure behaviours are only switched on at high densities (‘quorum’) [72]. Microbial pathogens are masters of coordinating collective actions; their quorum-sensing system enables density estimation via collectively produced diffusible molecules [72]. The recent discovery that malaria parasites secrete protein and DNA containing microvesicles that influence the sexual differentiation of other parasite cells [73,74] may be a mechanism to organize the density-dependent decisions observed in reproductive effort and sex allocation [21,75,76].

## 3. Virulence evolution

Parasites engage in clearly selfish acts with the hosts and vectors they exploit; here we give a brief overview of answers to the basic question of why parasites harm the very source of their livelihoods. The development of evolutionary theory to explain virulence (parasite-induced harm to the host) has a long history. Theories for the evolution of virulence can be categorized into four broad hypotheses [77,78] under which high virulence is attributed variously to: (i) novel host–parasite associations [79,80]; (ii) transmission–virulence trade-offs [81]; (iii) coincidental evolution of virulence factors [82,83]; and (iv) short-term within-host evolution [84]. For many infectious diseases, social interactions among parasites and virulence are coupled, but the nature of this relationship varies according to the type of interactions involved and who the interaction partners are.

**Figure 2.**
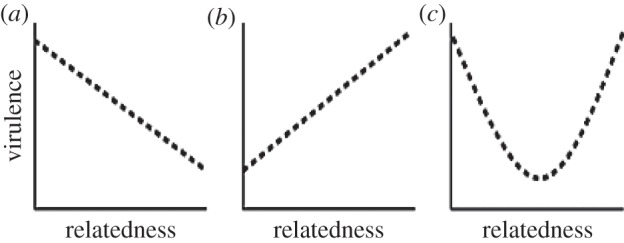
Theoretical relationships between virulence and relatedness under conditions of: (*a*) individual exploitation (virulence maximized at low relatedness) (*b*) collective exploitation (virulence maximized at high relatedness) (*c*) spiteful interactions, e.g. when harming competitors trades off against replication that causes virulence. (summarized by [16]).

The most influential theoretical framework for virulence evolution centres on virulence being maintained as a result of an unavoidable constraint linking the benefits of transmission with the costs of virulence. In this view, virulence (measured as host death) is an unavoidable cost of the host exploitation required for transmission to new hosts [85–88]. If the costs of increasing exploitation accelerate more rapidly than the transmission benefits of increasing exploitation, then natural selection favours an intermediate level of host exploitation (optimal virulence) [81,89]. Following from this premise, the relatedness of co-infecting parasite genotypes can modulate the best or evolutionary stable strategy of virulence, depending on the nature of social interactions among co-infecting parasites (figure 2). When co-infecting genotypes have direct control over their mechanisms of host exploitation, the benefits of increased exploitation are felt by the individuals responsible whereas the costs of virulence are shared by all, favouring greater virulence than that of parasites in single genotype infections [86–89]. By contrast, if co-infecting parasites work collectively to exploit the host (for example, via the secretion of shared extracellular digestive enzymes), then the benefits of exploitation become collectivized and mixed infections can select for ‘non-producer’ cheats that attenuate virulence [90]. In both scenarios, the spread of cheats (either over- or under-exploiters) undermines the productivity of the infection as a whole [56,88] but has opposite consequences for virulence.

## 4. Interactions in infections

The virulence–transmission trade-off models and their ‘virulence–kin-selection’ offshoots have been influential in the development of a vast body of subsequent theory [81]. Empirical testing has proceeded at a slower pace but only a few systems have provided support for the virulence–transmission trade-off [83,91]. A central theme emerging in the disease evolution literature is that within-host ecological dynamics are critical determinants of parasite sociality and so virulence [83,91,92]. In the following sections, we take a mechanistically inspired bottom-up approach, viewing virulence and transmission as emergent properties of complex within-host processes and highlighting five aspects of infections that can shape parasite social behaviours: (i) public goods, (ii) mobile elements, (iii) phenotypic plasticity, (iv) spatial structure and (v) multi-species interactions. We illustrate that a better understanding of these processes brings new perspectives to the traditional ‘top-down’ frameworks for the evolution and epidemiology of virulence and transmission.

### (a) Public goods

A central aspect of interactions between microbial pathogens is the collective engineering of their shared environment via the secretion of costly ‘public goods’ molecules. These molecules generate a range of benefits to any neighbouring cell that are suitably equipped to profit. For example, public goods molecules may scavenge for limiting resources (e.g. siderophores), aid in the construction of biofilms (e.g. adhesive polymers), kill competing lineages (e.g. bacteriocins) or enhance host exploitation (e.g. digestive enzymes, toxins). Because these molecules are individually costly to produce and yet return a collective benefit, they have become a focus in the study of bacterial cooperation. Among the best-studied model system for public goods cooperation is iron scavenging by secreted siderophores in the opportunistic bacterial pathogen *P. aeruginosa* (and related pseudomonads) [34,93,94]. *In vitro* studies in iron-limited environments have demonstrated that the fate of siderophore-producing ‘cooperator’ lineages in competition with non-producer ‘cheats’ is dependent on the degree of strain mixing or relatedness. When relatedness is high (each sub-population founded by a single clone), producers outcompete cheats, because the benefits of cooperation are disproportionately high for other cooperators. By contrast, when relatedness is low (e.g. each sub-population founded by multiple clones), cheats outcompete producers [34] (but see [95] for an exception driven by strong non-social selection).

The general applicability of a public goods framework for microbial interactions mediated by secreted factors has recently been called into question by Zhang & Rainey [96]. Here again the experimental focus was on siderophore-mediated interactions, where the authors illustrated that in certain standard laboratory experimental conditions (KB media), the production of siderophores is redundant and selected against. This result serves as a valuable reminder that the benefits of secreted molecules are undoubtedly environment dependent, and in this particular environment the secreted molecule does not provide benefits to neighbours and therefore does not function as a public good. Kummerli & Ross-Gillespie [97] responded to Zhang & Rainey [96] with an analysis of the iron content of KB, revealing that it is relatively iron-replete, ensuring that siderophore production is unlikely to provide sufficient benefit to merit the costs of production. From this, Kummerli & Ross-Gillespie conclude that there is no difficulty for the public goods framework, so long as the environmental context is adequately accounted for [97].

### (b) Mobile elements: infectious cooperation and locus-specific relatedness

The maintenance of cooperation via a single-cell bottleneck for each sub-population (as in [34] discussed earlier) is a very stringent condition. How is cooperation maintained under more realistic conditions that allow for some strain mixing, and more frequent interactions with cheats due to mutation or migration? The peculiar biology of bacteria points to an intriguing role played by their molecular parasites in maintaining the cooperative phenotypes of their bacterial hosts. Bacteria are prone to infection with a diverse array of molecular parasites that are able to spread infectiously via horizontal gene transfer (HGT) through a population, bringing novel genes along for the ride [98]. Key among these molecular parasites are plasmids, vectors of many medically significant alleles including antibiotic resistance and toxins [18]. Initial theoretical work suggested that the invasion of cheats into a population of cooperators could be prevented if the cooperative trait was encoded by an infectious conjugative plasmid [99]. In this scenario, cheats are liable to be re-programmed via infection with the cooperation-inducing plasmid. A key assumption of this model is that all plasmids carry the cooperative trait, so any act of infection will also increase cooperation, by hitch-hiking on the conjugation alleles.

But what if the social dilemma between cooperative and cheating alleles is played out at the level of the mobile element? More recent theory has pointed out that in an unstructured environment, ‘cheat’ plasmids will outcompete ‘cooperative’ plasmids for the same conditions that favour cheating chromosomal alleles over their cooperative rivals [100], because, again, the benefits of cooperation are not preferentially returned to cooperative alleles. However, the picture changes in structured populations, in which bacteria exploit discrete patches (e.g. hosts), linked by migration and/or transmission. Population structure introduces non-zero relatedness, and so the patterns of relatedness are now predicted to vary at different points of the genome depending on the rate of HGT [17,18,101]. Plasmids with high rates of HGT can readily copy themselves into neighbouring cells within a patch, and so if a cooperative plasmid gene generates benefits for neighbouring cells, it is now more likely to aid gene-copies in neighbouring cells to reproduce. In other words, highly conjugative plasmids gain a greater inclusive fitness return from helping neighbouring cells, favouring cooperative investments at these loci. Bio-informatic support for this inclusive fitness hypothesis has been demonstrated across 20 strains of *Escherichia coli*, where genes liable to experience greater HGT were more likely to code for secreted (cooperative) traits [17]. More recently, experiments show that HGT promotes plasmid-specific relatedness and selection for plasmid-encoded cooperation [102].

### (c) Phenotypic plasticity: adaptive adjustment of behaviours

An important feature of parasite lifestyles is that their social environments change constantly, and so parasites have evolved mechanisms to regulate their behaviours. HGT is a form of genetic plasticity that enables the loss and gain of locally adapted alleles [18], but parasites also excel at phenotypic plasticity, extracting multiple phenotypes from one genotype. Adaptive phenotypic plasticity—the ability of an organism to change its behaviour or morphology to fit the environment—is a ubiquitous solution to the challenges of life in a changing environment. Plasticity enables organisms to maintain fitness by altering their phenotype, through mechanisms such as differential gene expression, to best suit their circumstances [103], and here we focus on how plasticity in the behaviours (life-history traits) of parasites are shaped by their social environment within the host. For example, kin discrimination is a plastic response to social circumstances. By ensuring parasites only cooperate under conditions of high relatedness, kin discrimination may maintain cooperation by ensuring that the behaviour is adaptive from an inclusive fitness perspective, by limiting the potential to be exploited by cheats. Moreover, as well as enabling organisms to respond quickly once environmental change has occurred, organisms can also respond to predictors of future environmental change which enables appropriate phenotypes to be adopted in a timely manner [104].

Typically, evolutionary biologists and parasitologists have overlooked the notion that plasticity can produce qualitative and adaptive changes to the genotype-wide social phenotypes of parasites during infections. This is because they assume that parasite responses to environmental perturbation are mostly directed at maintaining homeostasis. As a result, variation in parasite behaviours is often—and potentially incorrectly—attributed to the footprint of host regulation rather than parasites making strategic decisions. For example, when the coordinated cell cycles of the rodent malaria parasite *Plasmodium chabaudi* are perturbed, they become rescheduled during infection and return to matching the host circadian rhythm. Whether parasite cell cycles are passively scheduled by host factors with a circadian basis or by parasites actively and collectively adjusting their timing is unclear [105]. However, evidence suggests that parasites are responsible for collectively coordinating their cell cycle schedules: synchronous and asynchronous malaria parasite species maintain their schedules in the same host environment (i.e. age–sex–strain-matched inbred mice); there are fitness benefits for parasites with cell cycles matched to the host circadian rhythm and matched infections cause greater virulence to the host [106,107]. Clearly, plasticity in parasite social behaviours complicates the understanding of within-host dynamics, but identifying to what extent parasite and/or host genes are responsible is central to interrogating their evolution.

**Figure 3.**
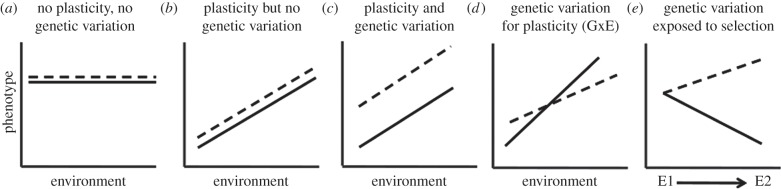
Phenotypic plasticity and reaction norms. In panel (*a*), phenotype does not vary with the environment and both genotypes have identical reaction norms. In panel (*b*) both genotypes are plastic and (*c*) there is also genetic variation. Panel (*d*) illustrates a genotype-by-environment interaction (G × E), where both genotypes are plastic but their phenotypic reaction norms vary. Genetic variation and G × E can complicate how much genetic variation is exposed to selection; in panel (*e*) the genotypes produce the same phenotype in environment (E) 1 but not in environment 2, so selection can only differentiate between the genotypes in environment 2.

The diversity of phenotypic plasticity in parasite social behaviours is illustrated in table 2. These traits are adjusted in response to social context and have consequences for virulence and transmission. Unfortunately, evolutionary theory has mostly ignored these behaviours, focusing instead on virulence. This is problematic because changes in virulence are achieved by changes in underlying traits (e.g. behaviours) expressed by both the host and parasites. As the social behaviours underpinning virulence and transmission are likely to be linked by genetic correlations (i.e. different traits are shaped by the same genes) and/or resource allocation trade-offs, the nature of these interactions is central to understanding and predicting virulence evolution [23]. Furthermore, when different genotypes respond to the environment in different ways (genotype-by-environment interactions or G × E), environmental change can expose (or hide) genetic variation in plasticity to natural selection [119] (figure 3). Ecological perturbations such as drugs, vaccines and host shifts are all candidate motors for constraining or facilitating evolution, depending on how the perturbation affects the amount of genetic variation underpinning parasite phenotypes. For example, genetic variation for sex ratio adjustment and reproductive effort in response to social context has been documented in malaria parasites [21,76] and these behaviours are determinants of how parasites survive drugs and overcome transmission-blocking immunity [75,120,121]. However, how G × E affects the speed that parasites could respond to selection on these behaviours is not known.

**Table 2.** Examples of phenotypic plasticity in parasite social behaviours. That phenotypes are a product of both genotypes and the environment, and how they interact, is well known, but often the environment is viewed as obscuring the connection between genes and phenotypes. However, how social behaviours are influenced by environmental variation matters because they affect virulence and transmission. Because multiple environmental factors change simultaneously during infections and virulence and transmission phenotypes are products of multiple social behaviours, parasites can produce a wide range of adaptive phenotypes faster by plasticity than when beneficial mutations or recombination are required to generate new phenotypes.

behaviour/ trait	what happens and why?
developmental schedules	In the host blood, cycles of asexual replication in many species of *Plasmodium* are tightly synchronized; individual parasites transit through each cell cycle stage and ultimately burst out of their red blood cells in unison and at particular times of day. The duration and synchronicity of cell cycles are plastic [105]. An adaptive basis of this plasticity is yet to be established but in-host competition and host immune responses are likely drivers [108]. Disrupted *P. chabaudi* schedules result in lower virulence (anaemia; [107]) but quiescence can also help *Plasmodium falciparum* tolerate antimalarial drugs [109].
lysis time	Pi 2 bacteriophage must lyse their bacterial host (*Pseudomonas fluorescens*) to transmit. They evolve a plastic lysis time in which they kill host cells more rapidly when co-infecting host cells with other phage than when infecting alone [38]. Plasticity in lysis time evolved in phage lines in mixed-infection conditions owing to the frequent variability in whether they encounter co- or single infections in this treatment (the lysis time in single-infection conditions did not change or become plastic in response to selection). This plasticity enhances the competitive ability of phage since non-plastic phage have fewer mature propagules upon cell lysis and suggests virulence and transmission differ according to whether parasites are in single or mixed genotype infections. In addition, lysis inhibition (LIN) is a mechanism of burst-size increase and latent period extension induced by T4 bacteriophage secondary adsorption of T4-infected *E. coli* cells. This plastic growth strategy is an adaptation to environments containing high densities of T4-infected cells [110]: when T4-infected cell density is high, high densities of free phages are generated, uninfected cells are rapidly infected, secondary adsorption is likely and LIN is induced with high probability [110–113].
public goods	The production of an iron-scavenging molecule (pyoverdin) by *P. aeruginosa* bacteria is a cooperative trait. Pyverdin production per bacterium is tightly regulated by the intracellular supply of free iron, leading to decreased *per capita* production at higher cell densities and increased production in the presence of non-producing cheats. This phenotypic plasticity significantly influences the costs and benefits of cooperation. Specifically, the investment of resources into pyoverdin production is reduced in iron-rich environments and at high cell densities, but increased under iron limitation, and when pyoverdin is exploited by cheats [114,115]. Regulatory control of public goods provisioning can further protect producers from exploitation by cheats by ‘metabolic prudence’, limiting production to environments where the relative costs of production are minimized [60]. More globally, the regulatory control of multiple secreted factors is under the control of quorum-sensing mechanisms in numerous bacteria, including several significant pathogens [116].
reproductive effort	*Plasmodium* must replicate asexually in the vertebrate host and undergo a round of sexual reproduction in the vector. This means that resources must be divided between growth (the production of asexual stages for in-host survival) and reproduction (the production of sexual stages for transmission). *P. chabaudi* adopts reproductive restraint when facing in-host competition, which is consistent with investing in asexual replication (a key determinant of competitive ability) to gain future transmission opportunities [20,76]. *P. falciparum* also adopts reproductive restraint in response to low doses of drugs, suggesting this is a general strategy for coping with stresses encountered in the host [75].
sex allocation	In addition to the growth versus reproduction trade-off described earlier, *Plasmodium* must also divide resources between male and female transmission stages (sex ratio). Sex ratios in *P. chabaudi* and *P. falciparum* are adjusted in response to the inbreeding rate, which is determined by the number of co-infecting genotypes and their relative frequencies [21]. In single infections, female-biased sex ratios maximize zygote production and increasing the proportion of males in mixed infections, especially if a weak competitor, maximizes representation in the zygote population.
suicide	A ‘suicide trait’ cannot be constitutively expressed (if everyone dies before reproducing, genes for the trait cannot be inherited). Thus, the proportion of parasites that die may be precisely adjusted in response to variation in the density and relatedness of co-infecting parasites, or noisy expression of the genes involved may ensure phenotypic variation [41,117]. The release of bacteriocins to kill competitors requires bacterial cells to lyse themselves in many species, including *E. coli* [14]. The benefits accruing to surviving kin are highest when at low density, but this is when the costs of losing group members are greatest. By contrast, *Plasmodium* experiences crowding in the vector: high parasite densities reduce per parasite productivity and elevate vector mortality so suicide in the stage infective to the vector is predicted to regulate infection intensity [118].

The potential for interactions between plasticity and evolution introduces yet more complications to understanding how social behaviours shape parasite fitness for two additional reasons. First, adaptive plasticity can facilitate parasite evolution simply by providing more time and/or individuals for beneficial mutations to arise because their survival is enhanced [122]. By contrast, when plasticity buffers parasites against the loss of fitness in a novel environment, the strength of selection imposed by environmental change is reduced, and so parasite evolution is constrained. Quantitative theory that makes testable predictions for the opposing effects of plasticity on rates of evolution is urgently needed. For example, the social behaviours of malaria parasites provide tolerance to drugs: plasticity in reproductive restraint helps buffer against the impact of drugs on within-host survival [75]. Thus, selection for other resistance traits (e.g. drug efflux pumps, alternative metabolic or detoxification pathways) is weakened but this clinically beneficial outcome may be undermined because the greater number of surviving parasites offers more opportunities for resistance mutations to occur. Second, while a behaviour may be plastically adjusted in response to social context, the consequences of the action can subsequently feedback to affect social context. For example, bacteriophage plastically speed-up their host lysis time phenotype in response to being in a mixed versus a single infection, i.e. they are responding to the social context encountered within their host [38]. By lysing the host cell before non-plastic phage can transmit, the plastic phage gain a competitive advantage and consequently become increasingly more likely to interact with related phage.

It is our view that incorporating phenotypic plasticity into social evolution theory represents a milestone for bringing theoretical work and empirical observations closer. To some extent, for a few social traits (e.g. sex allocation of malaria parasites [123]) existing theory that predicts what fixed traits should be at equilibrium can also apply to plastic strategies, and so can be used to make quantitative predictions. However, analyses that incorporate phenomena specific to plasticity, such as its costs and limits, are lacking. The costs and limits of plasticity matter because they may maintain genetic variation in natural populations [124] and could offer novel disease intervention targets [23]. The importance of the costs and limits of plasticity are illustrated by parasites for which the host is an infrequent environment. For example, *P. aeruginosa* is a supreme generalist microbe, able to grow in soil, water and diverse animal and plant hosts, thanks to high investment in regulatory factors [83,125]. While the benefits of extensive and complex regulatory control are easily appreciated in its broad host range, they also raise the potential cost of making ‘bad decisions’, turning on genes inappropriately when faced with a novel environment. During initial human colonization, *P. aeruginosa* turns on an array of virulence factors [126,127] that cause serious damage to the host. However, many of these damaging traits are subsequently lost or turned off during within-host evolution [128], suggesting that the initial plastic responses were maladaptive. It is possible that the loss of these secreted virulence factors is due to social interactions favouring non-producing ‘cheats’ that do not pay the cost of the collectively useful virulence traits [34,129]. However, the continued ability of these ‘cheat’ strains to persist [129] suggests that the virulence factors are redundant in the host lung, and their initial upregulation was a ‘bad decision’ [83].

### (d) Spatial structure

A major limitation of both theoretical and experimental work is that, for simplicity, historically most microbial (especially bacterial) studies considered well-mixed groups in liquid where local spatial structure is minimal [34,130]. This view may be a reasonable approximation for taxa like malaria parasites, where social interactions appear to play-out on a host-wide scale. However, hosts are not ‘a well-mixed bag’ of resources and immune defences, and so the reality for many parasites is that infections are far more structured at a local (within-host) scale [131]. For instance, many bacteria stick themselves to host surfaces or attach to each other, in groups called biofilms. Social interactions are most intense when individuals live side-by-side in these structured environments [132]. For example, conflict between cooperating and cheating *P. aeruginosa* is more intense in biofilms than in liquid culture [133]. In a biofilm, the presence of cheats causes a greater reduction in population growth, reduces the structural integrity of biofilms and increases susceptibility to antibiotics [133]. However, the advantages of life in a biofilm may be tempered by a trade-off recently observed in *Vibrio cholerae,* between the benefits of being better competitors within the host and the costs of impaired ability to disperse [134].

### (e) Multi-species interactions

Most natural parasite communities are characterized by spatial structure, a multitude of co-infecting species and several environments to cope with. For example, the lesson from bacterial metagenomics is that thousands of species are commonly present in any one environment [132,135]. By contrast, the primary focus of parasite social evolution studies has involved examining what happens when multiple genotypes of a single species are mixed (e.g. [21,37,76,136]). Cross-species parasite social interactions are diverse: depending on the species in question, an incoming species can by excluded, facilitated or unaffected by a resident species [137]. For example, an ongoing malaria infection can exclude conspecifics [138,139] but strongly facilitate infection by heterospecific malaria parasites. In the latter case, species preferentially infecting mature red blood cells generate anaemia to which the host responds by producing young red blood cells, which is predicted to facilitate malaria species that prefer the abundant young age class, resulting in far higher virulence than single-species infection [140]. However, the mechanisms that determine cross-species interactions are highly diverse, ranging from resource competition, interference competition (e.g. the production of antibiotics and bacteriocins), immune-mediated apparent competition and facilitation (e.g. cross-feeding on partner metabolic byproducts, immunomodulation) [141]. Together this menu of interactions contributes to the astounding diversity of communities of commensals, symbionts and parasites found within multicellular organisms.

A major challenge to unravelling the mechanisms underpinning how communities function is the necessity to combine molecular and ecological approaches to study highly complex assemblies. A measure of the scale of the problem can be seen by the emergent ecological complexity generated by a simple two-species interaction governed by a single mechanism of metabolic exchange—a food for detoxification exchange—where a cross-feeding partner relieves a producer lineage of by-product toxicity. Recent theory has demonstrated that this simple exchange can generate mutualistic, competitive and exploitative functional relationships, and diverse spatial patternings, dependent on the exact parametrization of the molecular exchange [142]. Unravelling the complexity of these interactions—and how they affect evolution—is urgently required because the microbial communities inside vectors are being manipulated to control disease [143].

## 5. Why the social lives of parasites matter

Parasitism is one of the most successful modes of life, as measured by how often it evolved and how many parasitic species are presently in existence [144]. Consequently, if explaining cooperation is one of the greatest problems for evolutionary biology, then explaining cooperation in parasites is one of the key aspects of this problem. The irreducible mishmash of proximate causality of social behaviours in traditionally studied animal taxa is far more accessible for parasites, thanks to their relatively simple and manipulatable genotype–phenotype maps. Parasites make excellent model organisms thanks to their short generation times; ability to generate some real-world complexity, even in the laboratory, by studying *in vivo* infections; and well-defined, measurable, social behaviours. Moreover, the applied importance of parasites has resulted in a vast resource of tools and literature on their molecular and cellular biology, so the genetic and molecular mechanisms that underlie social behaviours can be identified and precisely manipulated [8].

Incorporating a ‘bottom-up’ approach provides a novel perspective on the evolution and maintenance of parasite social behaviours and provides new opportunities for theory-led experimental testing. For example, by understanding aspects of interactions in infections such as those highlighted in this article, traditional virulence evolution theory may be better reconciled with data. Research has focused on social interactions between parasites within hosts (probably owing to the greater interest in disease pathology than transmission) and so social interactions inside vectors have been overlooked, but we expect that they are equally worthy of investigation. Moreover, for parasite species whose life cycles include multiple host species or periods in the abiotic environment, quantifying how social behaviours at these different scales integrate to shape parasite fitness is also a huge challenge, and highlights the need to consider within-host biology in its broader context.

The social behaviours of parasites contribute to virulence, transmission and resistance to anti-parasite drugs, as illustrated throughout the text and tables of this article. The field of ‘Darwinian Medicine’ aims to use ecological and evolutionary principles to inform the treatment of infections to ensure that interventions are as evolution-proof as possible, and prevent the evolution of more harmful parasites in response to anthropogenic pressures. ‘Hamiltonian Medicine’ is emerging as a subset of this endeavour, asking how parasite social systems and interactions might be subverted or manipulated to better control disease [9,145]. By recognizing that parasites rely on social behaviours to infect and transmit, novel strategies for treatment have been revealed (table 3).

**Table 3.** The potential of ‘Hamiltonian Medicine’: examples and limitations of proposed biomedical applications of parasite sociality.

concept	examples
cheat therapy	A strategy as simple as the introduction of a cheat (non-producer) strain can lead to direct reduction in parasite virulence, as well as a reduced bacterial population size, that may make the infection more susceptible to other intervention strategies. For example, the introduction of cheater mutants with reduced expression of secreted virulence factors into infections of the bacterial pathogen *P. aeruginosa* reduces mortality in a mouse model [146], at least in the case of simultaneous inoculation of the target wild-type and the cheater ‘treatment’. The ability of cheats to increase in frequency within a wild-type infection while simultaneously decreasing virulence has led to the idea of exploiting cheater invasion to introduce medically beneficial alleles into infections, such as sensitivity to antibiotics or a lethal toxin under the control of an inducible promoter, which when activated would eliminate both cooperators and cheats [19]. This approach resembles phage therapy, where a live and natural enemy is administered to control an infection at a specific site, and shares the benefits of responsive dosing (the treatment can amplify at the target site, unlike chemical therapeutics). However, cheat therapies face many of the obstacles we outline in the main text—they may be vulnerable to ‘reprogramming’ by cooperation-inducing plasmids, they may be unable to exploit established cooperator populations owing to within-host structure, or owing to plastic phenotypic changes in the resident. Finally, rare cheats may be unable to overcome the local-adaptation advantages of established wild-type infections [95,147].
drug resistance	Drug resistance mechanisms are often thought to impose fitness costs in the absence of drugs. Experiments using malaria parasites suggest that these fitness costs include competitive inferiority, and so suppression by wild-type genotypes in mixed infections could constrain the spread of resistance [148]. However, the extent to which suppression impacts on resistance in natural infections and how this could interact with eradication programmes is unclear. This is because as parasite prevalence decreases, infections will increasingly contain highly related parasites, which are more likely to cooperate than compete. Traditional antibiotics act by killing or stopping cell division, and resistant mutants rapidly replace the original susceptible strains. Instead, if a drug attacks a cell's ability to secrete a public good that contributes to virulence (an ‘anti-virulence’ drug), then resistant mutants that re-evolve secretion will promote the growth of susceptible cells around them, reducing the spread of resistance. Moreover, because the susceptible cells do not pay the cost of secretion (i.e. they cheat), this puts resistant parasites at a competitive disadvantage, further reducing the spread of resistance [148–151].
evolutionary traps	An underexplored avenue concerns manipulating parasite kin recognition and communication systems to ‘trick’ parasites into adopting strategies that are suboptimal for their fitness and of clinical or epidemiological benefit. Evolving resistance to this type of intervention could be difficult because solutions would probably involve losing the benefit of coordinated action in untreated infections. For example, in malaria parasites, investment in asexual stages (which are responsible for disease symptoms) versus sexual stages is plastic. Parasites competing in mixed infections invest relatively less in sexual stages than when in single infections [76]. A drug that mimics being in a single infection (e.g. masks the cues of competition), and so induces parasites to invest more in sexual stages, will result in less virulent infections, and as long as conditions are vector-free there will be no increase in the risk of transmission to other hosts. Furthermore, the additional sexual stages will provide a stronger stimulus to the host immune system and the resulting responses could more effectively block the transmission of future malaria infections [152]. An approach to blocking transmission would be to induce mass suicide in the vector [15].

## 6. Conclusion

A key strength of evolutionary biology is that theory is used to motivate experiments. Historically, this has been the case, with many empirical tests stemming from the basic virulence–transmission trade-off models and their ‘virulence–kin-selection’ offshoots. However, for topics such as phenotypic plasticity, empirical work is often ahead of social evolution theory and this disconnect is especially apparent in systems that have applied importance. We recognize that the complexity of within-host parasite ecology may have been off-putting for evolutionary theorists since, on the face of it, generalities seem unlikely and explaining what is going on requires deeper knowledge of the biological details of individual study systems. However, generalities do exist—such as public goods, mobile elements, phenotypic plasticity, within-host spatial structure and multi-species interactions—that will provide rewarding avenues for future theoretical and experimental research.
